# Clinical outcomes of pomalidomide‐based and daratumumab‐based therapies in patients with relapsed/refractory multiple myeloma: A real‐world cohort study in China

**DOI:** 10.1002/cam4.7232

**Published:** 2024-05-02

**Authors:** Xiaoyan Han, Xincheng Jiang, Jingsong He, Gaofeng Zheng, Yaqin Xiong, Yanling Wen, Yang Yang, Donghua He, Qingxiao Chen, Yi Zhao, Yi Li, Wenjun Wu, Zhen Cai

**Affiliations:** ^1^ Department of Hematology and Bone Marrow Transplantation Center The First Affiliated Hospital, School of Medicine, Zhejiang University Hangzhou Zhejiang China; ^2^ Institute of Hematology, Zhejiang University Hangzhou Zhejiang China

**Keywords:** daratumumab, multiple myeloma, pomalidomide, recurrence, refractory

## Abstract

**Background:**

Comparative investigations evaluating the efficacy of pomalidomide‐based (Pom‐based) versus daratumumab‐based (Dara‐based) therapies in patients with relapsed/refractory multiple myeloma (RRMM) remain scarce, both in randomized controlled trials and real‐world studies.

**Methods:**

This retrospective cohort study included 140 RRMM patients treated with Pom‐based or Dara‐based or a combination of pomalidomide and daratumumab (DPd) regimens in a Chinese tertiary hospital between December 2018 and July 2023.

**Results:**

The overall response rates (ORR) for Pom‐based (*n* = 48), Dara‐based (*n* = 68), and DPd (*n* = 24) groups were 57.8%, 84.6%, and 75.0%, respectively (*p* = 0.007). At data cutoff on August 1, 2023, the median progression‐free survival (PFS) was 5.7 months (95% CI: 5.0–6.5) for the Pom‐based group, 10.5 months (5.2–15.8) for the Dara‐based group, and 6.7 months (4.0–9.3) for the DPd group (*p =* 0.056). Multivariate analysis identified treatment regimens (Dara‐based vs. Pom‐based, DPd vs. Pom‐based) and Eastern Cooperative Oncology Group performance status (ECOG PS) as independent prognostic factors for PFS. In the subgroups of patients aged >65 years, with ECOG PS ≥2, lines of therapy ≥2, extramedullary disease or double‐refractory disease (refractory to both lenalidomide and proteasome inhibitors), the superiority of Dara‐based regimens over Pom‐based regimens was not evident. A higher incidence of infections was observed in patients receiving Dara‐based and DPd regimens (Pom‐based 39.6% vs. Dara‐based 64.7% vs. DPd 70.8%, *p =* 0.009).

**Conclusions:**

In real‐world settings, Pom‐based, Dara‐based, and DPd therapies exhibited favorable efficacy in patients with RRMM. Dara‐based therapy yielded superior clinical response and PFS compared to Pom‐based therapy.

## 1 INTRODUCTION

Multiple myeloma (MM), as a plasma cell malignancy, remains largely incurable, and the majority of patients eventually progress to relapse and/or refractory MM (RRMM).^1^ Within the past two decades, the availability of multiple novel therapeutic drugs has largely improved the outcomes of RRMM.^2^ Pomalidomide (Pom) is a third‐generation immunomodulatory agent, whose antimyeloma mechanisms mainly involve it binding to cereblon (CRBN), promoting the ubiquitination and degradation of IL‐2 transcriptional repressors, Ikaros and Aiolos, and negatively regulating transcriptional factors IRF4 and MYC.^3^ In 2013, Pom was approved for the treatment of patients with RRMM who had undergone at least two prior therapies, including lenalidomide and bortezomib.^4^ Daratumumab (Dara) is another representative drug that is widely available and used for the treatment of RRMM. As a human IgGκ monoclonal antibody, Dara directly targets the highly expressed CD38 antigen on plasma cells and induces their death. It also has immunomodulatory and immune‐mediated effects.^5^, ^6^ Dara has been approved for use as monotherapy or in combination with standard‐of‐care regimens for RRMM.

Following the approval of novel antimyeloma drugs and therapeutic combinations, the treatment paradigm for MM has rapidly evolved. Lenalidomide combined with bortezomib and a steroid has emerged as a predominant first‐line treatment; hence, patients relapsed often have had prior exposure or become refractory to lenalidomide and/or bortezomib.^7^ In the phase III OPTIMISMM trial, Pom combined with bortezomib and dexamethasone produced a median progression‐free survival (PFS) of 11.2 months in patients previously exposed to lenalidomide (71% lenalidomide refractory).^8^ In the CASTOR and POLLUX trials, the PFS benefit conferred by Dara in combination with bortezomib/lenalidomide and dexamethasone over bortezomib/lenalidomide and dexamethasone alone was maintained in patients given prior lenalidomide or bortezomib treatment.^9^, ^10^ The APOLLO trial assessed combinations of Dara, Pom, and dexamethasone (DPd) in patients who had received lenalidomide and a proteasome inhibitor (PI) (79% lenalidomide refractory, 47% PI refractory), and a median PFS of 12.4 months was reported.^11^ Based on the demonstrated benefits in RRMM patients, including those previously treated with or refractory to lenalidomide and/or bortezomib, Pom‐based, Dara‐based, and DPd regimens have become prevalent salvage options after the failure of front‐line therapies.^12^

Even with substantial evidence regarding specific regimens, the absence of comparisons between these regimens creates a challenge for physicians and patients in the treatment options for RRMM. Moreover, in view of the variation in baseline characteristics among patients, including age, frailty, comorbidities, disease biology, and treatment history, determining the optimal sequence and combination of drugs for distinct populations represents an outstanding clinical challenge.

This retrospective study reports the efficacy and safety of Pom‐based, Dara‐based, and DPd regimens for patients with RRMM in a single Chinese tertiary hospital. Comparisons were also made of the response and survival rates between these regimens across distinct subgroups.

## 2 METHODS

### 2.1 Study populations

This study was a single‐centered, retrospective cohort study involving 140 patients at the First Affiliated Hospital, Zhejiang University School of Medicine, Hangzhou, Zhejiang, China, conducted between December 2018 and July 2023. The study inclusion criteria were as follows: age ≥18 years; diagnosis of MM based on International Myeloma Working Group (IMWG) criteria; the presence of measurable disease; 1–5 previous lines of therapy (LOT), and either relapsed or refractory to the last LOT; relatively complete clinical data; and completion of at least one cycle of Pom/Dara‐based treatment. Patients with newly diagnosed MM, amyloidosis, primary plasma cell leukemia, or solitary plasmacytoma were excluded. Patients were divided into three groups based on the treatment namely a Pom‐based group, a Dara‐based group, and a DPd group. When patients received more than one of the aforementioned regimens successively, they were categorized based on the regimen first received. The follow‐up endpoint was the date of the last follow‐up or death through August 1, 2023. Their demographics, disease characteristics, and clinical outcomes were extracted from the hospital electronic medical records database after the approval of the review board. This research was approved by the institutional ethics committee of the First Affiliated Hospital of Zhejiang University School of Medicine. Data collection included patients' demographics, hematological disease characteristics, treatment history, treatment course, response, relapse, survival, and adverse events (AEs).

### 2.2 Treatment

In this study, 48 patients received Pom‐based therapy which included bortezomib + Pom + dexamethasone (VPd; *n* = 24), ixazomib + Pom + dexamethasone (IPd; *n* = 12), Pom + cyclophosphamide + dexamethasone (PCd; *n* = 6), and Pom + dexamethasone (Pd; *n* = 6). Pom was administered orally with 2–4 mg as the starting dose (modified according to patient tolerance) once daily on Days 1–21 of 1 cycle (1 cycle was 28 days). Sixty‐eight patients were treated with Dara‐based therapies, mainly including Dara + bortezomib + dexamethasone (DVd; *n* = 27), Dara + lenalidomide + dexamethasone (DRd; *n* = 23), and Dara + selinexor + dexamethasone (D‐selinexor‐d; *n* = 17). Patients received intravenous Dara (16 mg per kg of body weight) weekly during cycles 1–2, every 2 weeks during cycles 3–6, and every 4 weeks thereafter until disease progression (PD) or unacceptable toxicity was reported. Twenty‐four patients received DPd therapy, among which 22 received DPd and 2 received Dara + bortezomib + Pom + dexamethasone (DVPd). The dose of Pom and Dara in the DPd group was consistent with the Pom‐based and Dara‐based groups. Oral dexamethasone was administered 40 mg (20 mg for patients aged ≥75 years) once daily on Days 1, 8, 15, and 22 of each cycle. The details of these regimens are presented in Table S1. Supportive care (e.g., bone protection [bisphosphonate or denosumab], renal function protection [hydration and alkalization]), prophylaxis, and management of AEs were provided according to the physician's decision.

### 2.3 Outcomes

The primary endpoints were PFS and the overall response rate (ORR). PFS was defined as the time from the initiation of Pom/Dara‐based therapies to the date of PD or death. ORR was calculated as the proportion of patients who achieved at least a partial response. Secondary endpoints included overall survival (OS, the time from the initiation of Pom/Dara‐based therapies to the date of death from any cause), time to next treatment (TTNT, the time from the initiation of Pom/Dara‐based therapies to the date of initiating the next line of antimyeloma treatment or the date of death), and safety (AEs). Hematological responses were evaluated according to IMWG response criteria. High‐risk cytogenetics was defined as the presence of del(17p), high‐risk IgH translocation [t(14;16) or t(14;20)], or amplification of 1q using fluorescence in situ hybridization (FISH). Minimal residual disease (MRD) was detected by multicolor flow cytometry at a threshold of 1 tumor cell per 10^4^–10^5^ white cells. AEs were assessed using the National Cancer Institute's common terminology criteria for adverse events (CTCAE) version 5.0. Efficacy assessments were conducted every 1–2 cycles or if a suspected relapse occurred. Optimal response data were considered as the outcome. Dose adjustments/interruptions and their causes during treatment were carefully recorded, and AEs were monitored throughout the treatment cycles.

### 2.4 Statistical analysis

Categorical variables are given as numbers and percentages (*n*, %) and comparisons made between groups using a chi‐squared test or Fisher's exact probability test, as appropriate. Continuous variables are presented as the median and range (median, range), and nonparametric tests were applied to look for differences between groups. Time‐to‐event data were analyzed using the Kaplan–Meier method and described by the median time‐to‐event and 95% confidence intervals (median, 95% CI). Time to follow‐up was estimated by the reverse Kaplan–Meier method. Patients who were lost to follow‐up were censored from the date of their final follow‐up. Differences between groups were evaluated using log‐rank tests. Univariate and multivariate logistic regression and Cox regression models for ORR and PFS were built to improve the balance of confounders between groups. Only covariates with *p* < 0.1 were entered into the multivariate model. The results are shown as an odds ratio (OR) or hazard ratio (HR) with 95% CIs. Exploratory subgroup analysis and interaction tests were performed for ORR and PFS, stratified by age, extramedullary infiltration, the international staging system (ISS) stage, cytogenetics, renal function, Eastern Cooperative Oncology Group performance status (ECOG PS), prior LOT and refractory status to lenalidomide, PI, and both lenalidomide and PI (double‐refractory). The results are presented as a risk ratio (RR) and HR with 95% CIs. Two‐sided *p* ≤ 0.05 was considered to be a statistically significant difference. Data analyses were performed using GraphPad Prism ver. 9.0 (GraphPad Software, San Diego, CA, USA) and SPSS ver. 26.0 (SPSS, IBM Corp., Armonk, NY, USA).

## 3 RESULTS

### 3.1 Baseline characteristics and treatment history of patients

The median age of our cohort was 63.5 years (range: 40–90), and 67/140 (47.9%) of the patients were identified as having high‐risk cytogenetics. Patients had received a median of 2 (1–5) LOTs before initiating Pom/Dara‐based therapies. Most had been previously treated with lenalidomide or PIs (87.1% and 100%), and the proportion of patients refractory to lenalidomide, PI or both lenalidomide and PI was 72.1%, 70.0%, and 51.4%, respectively. The baseline characteristics and treatment history of patients are shown in Tables 1 and 2. A higher proportion of patients in the DPd cohort were refractory to PI compared to the other cohorts (*p* = 0.03). Other baseline and prior treatment characteristics were similar across the three treatment groups.

**TABLE 1:** Baseline characteristics of patients.

Patient characteristics	Pom‐based (*n* = 48)	Dara‐based (*n* = 68)	DPd (*n* = 24)	*p*‐value
Age				
Median (range) in years	63.5 (49–79)	63.5 (40–90)	63.5 (40–81)	0.98
>65	20 (41.7)	31 (45.6)	11 (45.8)	0.90
Sex				
Male	27 (56.3)	43 (63.2)	14 (58.3)	0.74
Subtype				
IgA	12 (25.0)	11 (16.2)	8 (33.3)	0.43
IgG	20 (41.7)	35 (51.5)	7 (29.2)	
Light chain	10 (20.8)	13 (19.1)	7 (29.2)	
Others^a^	6 (12.5)	9 (13.2)	2 (8.3)	
Extramedullary disease				
Yes	11 (22.9)	21 (30.9)	4 (16.7)	0.34
DS				
III	39 (81.3)	51 (75.0)	19 (79.2)	0.72
ISS				
III	16 (33.3)	31 (45.6)	9 (37.5)	0.40
Cytogenetics				
High risk	24 (50.0)	29 (42.6)	14 (58.3)	0.39
No high risk or NA	24 (50.0)	39 (57.4)	10 (41.7)	
R‐ISS				
I + II	37 (77.1)	50 (73.5)	18 (75.0)	0.72
III	6 (12.5)	9 (13.2)	5 (20.8)	
NA	5 (10.4)	9 (13.2)	1 (4.2)	
eGFR				
Median (range) in mL/min	71.5 (4–123)	86 (7–136)	88 (8–120)	0.33
<60 mL/min	15 (31.3)	17 (25.0)	7 (29.2)	0.75
ECOG PS				
≥2	7 (14.6)	19 (27.9)	9 (37.5)	0.08
Years from diagnosis to the initiation of Pom/Dara‐based therapy				
≥3	25 (52.1)	24 (35.3)	10 (41.7)	0.20

*Note*: Data are shown as the median (range) and *n* (%).

Abbreviations: Dara‐based, daratumumab‐based; DPd, daratumumab plus pomalidomide and dexamethasone; DS, Durie‐Salmon Staging System; ECOG PS, Eastern Cooperative Oncology Group performance status; eGFR, estimated glomerular filtration rate by Cockcroft‐Gault equation; ISS, International Staging System; NA, not available; Pom‐based, pomalidomide‐based; R‐ISS, Revised‐International Staging System.

^a^ Include IgD (*n* = 14) and non‐secretory subtype (*n* = 3).

**TABLE 2:** Treatment history of patients.

Treatment history	Pom‐based (*n* = 48)	Dara‐based (*n* = 68)	DPd (*n* = 24)	*p‐*value
Prior lines of therapy, median (range)	2.0 (1–5)	2.0 (1–4)	2.0 (1–5)	0.20
Previous treatment				
Prior ASCT	9 (18.8)	11 (16.2)	7 (29.2)	0.38
Prior CAR‐T	5 (10.4)	1 (1.5)	2 (8.3)	0.10
Prior lenalidomide	43 (89.6)	57 (83.8)	22 (91.7)	0.51
Prior PIs	48 (100.0)	68 (100.0)	24 (100.0)	
Refractory status				
Lenalidomide	37 (77.1)	45 (66.2)	19 (79.2)	0.31
PIs	30 (62.5)	46 (67.6)	22 (91.7)	**0.03** ^a^ ^,*^
Both lenalidomide and PIs	25 (52.1)	30 (44.1)	17 (70.8)	0.08

*Note*: Data are shown as the median (range) and *n* (%). The footnote cue ‘*’ denotes *p*‐value ≤ 0.05. *p*‐value ≤ 0.05 are shown in bold font.

Abbreviations: ASCT, autologous stem cell transplantation; CAR‐T, chimeric antigen receptor (CAR) T‐cell therapy; Dara‐based, daratumumab‐based; DPd, daratumumab plus pomalidomide and dexamethasone; PIs, proteasome inhibitors (such as bortezomib, carfilzomib, ixazomib); Pom‐based, pomalidomide‐based.

^a^ Pom‐based vs. Dara‐based, *p* = 0.57; Pom‐based vs. DPd, *p* = 0.009.

### 3.2 Treatment course

At the time of data cutoff (August 1, 2023), the median follow‐up period was 17.7 months (95% CI: 13.5–21.9) for the Pom‐based group, 12.3 months (11.3–13.3) for the Dara‐based group, and 18.4 months (13.3–23.6) for the DPd group (*p =* 0.98).

The median number of treatment cycles was 4.5 (range: 1–19) in the Pom‐based group, 4 (1–48) in the Dara‐based group, and 5 (1–18) in the DPd group. A total of 72.1% (101/140) patients discontinued treatment because of PD (Pom‐based 32, Dara‐based 30, DPd 12), AEs (Pom‐based 4, Dara‐based 4, DPd 3), switching to chimeric antigen receptor T‐cell therapy (Pom‐based 1) or autologous stem cell transplantation (Dara‐based 7, DPd 1), personal choice (Pom‐based 2, Dara‐based 1), and physician's decision (Pom‐based 1, Dara‐based 2, DPd 1). Twenty‐two (22/72, 30.6%) patients required modification of the Pom starting dose based on AEs (Pom‐based 10, DPd 12). Dose reductions and interruptions of Pom during treatment were reported in 9 (12.5%) patients (Pom‐based 4, DPd 5) and 37 (51.4%) patients (Pom‐based 20, DPd 17), due to safety or economic concerns. Forty‐six (46/92, 50.0%) patients underwent Dara temporary interruptions (Dara‐based 31, DPd 15) mostly due to AEs. During the follow‐up period, 27 (19.3%) patients had died (Pom‐based 12, Dara‐based 8, DPd 7), with the major cause of death being PD. Four (4/48, 8.3%) patients continued to receive Pom‐based regimens as maintenance therapy, while 8 (8/68, 11.8%) received Dara‐based regimens. In the DPd group, 5 (5/24, 20.8%) patients continued to receive the same regimen as maintenance therapy.

### 3.3 Clinical outcomes

**FIGURE 1:**
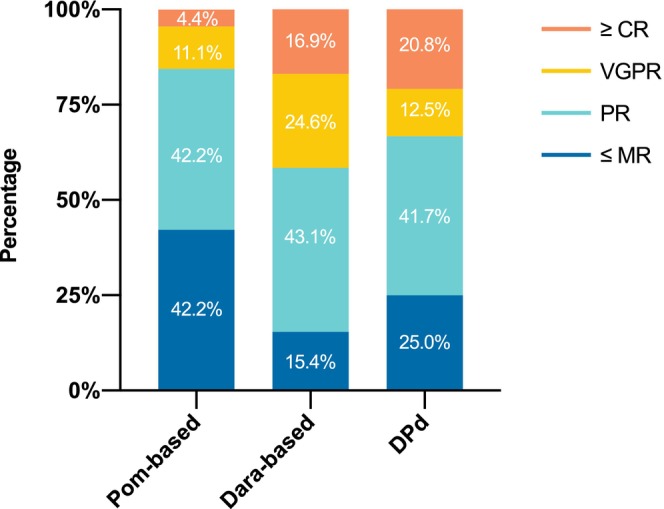
Response rates by regimens. CR, complete response; Dara‐based, daratumumab‐based; DPd, daratumumab plus pomalidomide and dexamethasone; MR, minimal response; Pom‐based, pomalidomide‐based; PR, partial response; VGPR, very good partial response.

As shown in Figure 1 and Table S2, notable differences in ORR (Pom‐based 57.8% vs. Dara‐based 84.6% vs. DPd 75.0%, *p =* 0.007) and ≥very good partial response (VGPR) rate (15.6% vs. 41.5% vs. 33.3%, *p =* 0.02) were discerned between the three treatment groups. The proportions of patients achieving an overall response (84.6% vs. 57.8%, *p =* 0.002) and ≥VGPR (41.5% vs. 15.6%, *p =* 0.004) were significantly increased in the Dara‐based group compared to the Pom‐based group. The DPd group also appeared to have a numerically superior ORR (75.0% vs. 57.8%, *p =* 0.16) and ≥ VGPR rate (33.3% vs. 15.6%, *p =* 0.09) relative to the Pom‐based group, but the apparent differences did not reach statistical significance. A difference in the complete or stringent response (≥complete response [CR]) rate among the cohorts was not obvious (Pom‐based 4.4% vs. Dara‐based 16.9% vs. DPd 20.8%, *p =* 0.09). Among the 18 patients who achieved a CR or better, one‐third received assessments for MRD, and 5 of these reached a negative status (Pom‐based 0/1, Dara‐based 4/4, DPd 1/1).

**FIGURE 2:**
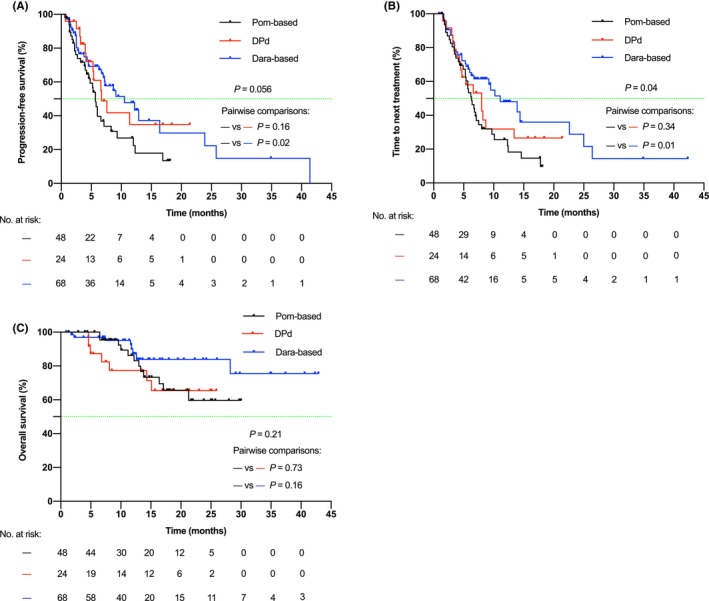
Kaplan–Meier curves for PFS (2A), TTNT (2B), and OS (2C) of Pom‐based, Dara‐based, and DPd groups. Log‐rank tests were used to evaluate differences between groups. Dara‐based, daratumumab‐based; DPd, daratumumab + pomalidomide and dexamethasone; Pom‐based, pomalidomide‐based.

The median PFS of patients was 5.7 months (95% CI: 5.0–6.5) in the Pom‐based group, 10.5 months (5.2–15.8) in the Dara‐based group, and 6.7 months (4.0–9.3) in the DPd group (*p* = 0.056, Figure 2A). In addition, the median TTNT was 6.3 months (5.1–7.5), 11.1 months (6.7–15.6), and 8.0 months (4.4–11.6) in the Pom‐based group, Dara‐based group, and DPd group (*p* = 0.04, Figure 2B). Upon the final analysis date, the median OS was not reached for any of the three groups (*p =* 0.21, Figure 2C).

To identify differences based on treatment outcomes, we performed univariate and multivariate analyses for ORR and PFS (Table 3 and Table S3). After adjusting for baseline confounders with *p* < 0.1 (ECOG PS and lenalidomide‐refractory status), treatment regimens (Dara‐based vs. Pom‐based, DPd vs. Pom‐based) and ECOG PS remained as independent predictors for PFS. In the multivariate model for ORR, no covariates with *p* < 0.1 were entered (Table S3).

**TABLE 3:** Univariate and multivariate analysis HR for PFS.

Parameter	Univariate HR (95% CI)	*p*‐value	Multivariate HR (95% CI)	*p‐*value
Treatment (reference Pom‐based group)				
Dara‐based	0.56 (0.34–0.93)	0.02*	0.46 (0.27–0.79)	0.004**
DPd	0.61 (0.31–1.18)	0.14	0.48 (0.24–0.95)	0.04*
ECOG PS (0–1 vs. ≥2)	1.96 (1.17–3.30)	0.01*	2.49 (1.43–4.31)	0.001***
Lenalidomide refractory (no vs. yes)	1.71 (0.97–2.99)	0.06	1.06 (0.50–2.23)	0.88

*Note*: Only covariates with *p* < 0.1 in the univariate model are outlined. HR (95% CI) has been rounded to the nearest hundredth. The footnote cue ‘*’ denotes *p*‐value ≤ 0.05, ‘**’ denotes *p*‐value ≤ 0.01, ‘***’ denotes *p*‐value ≤ 0.001. *p*‐value ≤ 0.05 are shown in bold font.

Abbreviations: CI, confidence interval; Dara‐based, daratumumab‐based; DPd, daratumumab plus pomalidomide and dexamethasone; HR, hazard ratio; Pom‐based, pomalidomide‐based.

**FIGURE 3:**
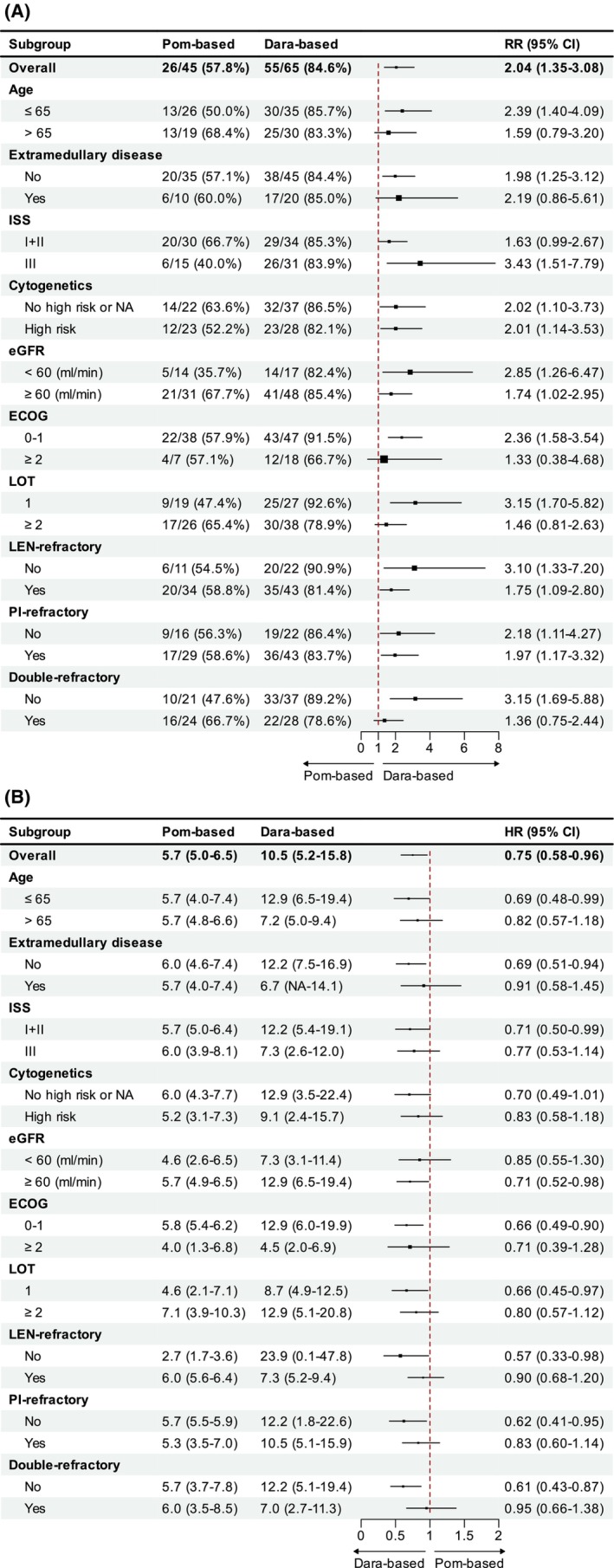
Post hoc subgroup analysis for ORR (3A) and PFS (3B). ORR are shown as number/patients (%), and PFS are shown as median (95% CI). Unadjusted results are presented. Dara‐based, daratumumab‐based; DPd, daratumumab plus pomalidomide and dexamethasone; ECOG PS, Eastern Cooperative Oncology Group performance status; eGFR, estimated glomerular filtration rate by Cockcroft‐Gault equation; HR, hazard ratio; ISS, International Staging System; LOT, line of therapy; NA, not available; PI, proteasome inhibitor; Pom‐based, pomalidomide‐based; RR, risk ratio.

The results of post hoc subgroup analysis of PFS and ORR based on important clinical characteristics are summarized in Figure 3. Due to the small sample size, subgroup analysis was not performed for the DPd group. In the comparison of the Dara‐based and Pom‐based groups, a consistent improvement in the overall response was observed in most subgroups, especially in patients who were aged under 65 years, without extramedullary infiltration, at ISS stage of III, ECOG PS of 0–1, non‐double‐refractory, and first relapse (Figure 3A). Moreover, patients in several subgroups showed a PFS improvement, including those ≤65 years, without extramedullary disease, at ISS stage I and II, without renal insufficiency, at ECOG PS of 0–1, first relapse, and who were not PI, lenalidomide or double‐refractory (Figure 3B).

### 3.4 Safety

Table 4 summarizes grade 3/4 (Gr 3/4) hematological and non‐hematological AEs regardless of attribution. In general, serious hematological AEs such as neutropenia, anemia, and thrombocytopenia were reported in 74 (52.9% ≥ Gr3), 60 (42.9% ≥ Gr3), and 48 (34.3% ≥ Gr3) patients, respectively. The most common severe hematologic toxicity observed was neutropenia. Infections and fatigue were common non‐hematological AEs, reported in 80 (57.1%) and 62 (44.3%) patients. Pneumonia was the predominant infection, occurring in 48 (34.3%) patients. Other AEs of special interest included peripheral neuropathy grade 3/4 (11, 7.9%), the infusion‐related reaction of Dara (14/92, 15.2%), *Herpes zoster* (3, 2.1%), and thromboembolic event (8, 5.7%). In contrast to the Pom‐based group, the incidence of infections was comparatively higher in patients treated with Dara‐based and DPd regimens (Pom‐based 39.6% vs. Dara‐based 64.7% vs. DPd 70.8%, *p =* 0.009). The DPd cohort required relatively frequent dose modifications and interruptions due to AEs (*p* < 0.001 and *p =* 0.02, respectively).

**TABLE 4:** Adverse events during treatment.

AEs	Pom‐based (*n* = 48)	Dara‐based (*n* = 68)	DPd (*n* = 24)	*p*‐value
Grade 3/4 hematological AEs				
Neutropenia	23 (47.9)	33 (48.5)	18 (75.0)	0.06
Anemia	16 (33.3)	29 (42.6)	15 (62.5)	0.06
Thrombocytopenia	14 (29.2)	25 (36.8)	9 (37.5)	0.65
Non‐hematological AEs				
Peripheral neuropathy grade 3/4	3 (6.3)	8 (11.8)	0 (0.0)	0.19
Diarrhea	3 (6.3)	11 (16.2)	2 (8.3)	0.22
Constipation	3 (6.3)	5 (7.4)	2 (8.3)	1.00
Infusion‐related reaction (daratumumab)	NA	9 (13.2)	5 (20.8)	NA
Infections	19 (39.6)	44 (64.7)	17 (70.8)	**0.009** ^a^ ^,**^
Pneumonia	15 (31.3)	20 (29.4)	13 (54.2)	0.08
Fatigue	18 (37.5)	29 (42.6)	15 (62.5)	0.12
Edema	3 (6.3)	5 (7.4)	2 (8.3)	1.00
*Herpes zoster*	1 (2.1)	0 (0.0)	2 (8.3)	**0.03** ^b^ ^,*^
Other rash	3 (6.3)	3 (4.4)	3 (12.5)	0.34
Thromboembolic event	1 (2.1)	6 (8.8)	1 (4.2)	0.39
Dose reduction (Pom/Dara) by AEs	14 (29.2)	0 (0.0)	17 (70.8)	<0.001***
Dose interruption (Pom/Dara) by AEs	15 (31.3)	28 (41.2)	16 (66.7)	**0.02** ^c^ ^,*^

*Note*: Data are shown as *n* (%). The footnote cue ‘*’ denotes *p*‐value ≤ 0.05, ‘**’ denotes *p*‐value ≤ 0.01, ‘***’ denotes *p*‐value ≤ 0.001. *p*‐value ≤ 0.05 are shown in bold font.

Abbreviations: AEs, adverse events; Dara‐based, daratumumab‐based; DPd, daratumumab plus pomalidomide and dexamethasone; Pom‐based, pomalidomide‐based. NA, not available.

^a^ Pom‐based vs. Dara‐based, *p* = 0.007; Pom‐based vs. DPd, *p =* 0.02.

^b^ Pom‐based vs. Dara‐based, *p* = 0.28; Pom‐based vs. DPd, *p* = 0.04.

^c^ Pom‐based vs. Dara‐based, *p* = 0.28; Pom‐based vs. DPd, *p* = 0.004.

## 4 DISCUSSION

The efficacy of Pom combination drug regimens has been extensively evaluated through numerous randomized controlled trials (Table 5). A meta‐analysis of 35 phase 2 and 3 clinical trials that involved Pom‐based doublet and triplet regimens, majorly including Pom/low‐dose dexamethasone (LoDex), bortezomib + Pom/LoDex, Dara + Pom/LoDex. The pooled data showed an ORR of 47.1% across all Pom‐based regimens.^36^ Real‐world studies in the United Kingdom,^37^ Poland,^38^ India,^39^ and Greece^40^ also reported the promising clinical effectiveness of Pom‐based regimens, with ORR ranging from 39.1% to 58.7% and the median PFS ranging from 5.2 months to 10.5 months. Our cohort represents a real‐world Chinese patient population, most of whom had previous treatment (87.1% and 100%) and were refractory (72.1% and 70.0%) to lenalidomide or PIs. An ORR of 57.8% and median PFS of 5.7 months were identified when Pom‐based regimens were administered after a median of two lines of treatment, a finding in good agreement with prior publications.

**TABLE 5:** Major clinical trials of Pom‐based, Dara‐based, and DPd regimens in RRMM.

Study reference	Phase	Regimen	Sample size	Study population	Median follow‐up (months)	Efficacy outcomes		
						ORR (%)	Median PFS (months)	Median OS (months)
Pom‐based								
**NIMBUS** Miguel et al., 2013^13^	III	Pd vs. high‐dose d	302 vs. 153	RRMM after ≥2 LOTs	10	31 vs. 10	4.0 vs. 1.9 (*p* < 0.0001)	12.7 vs. 8.1 (*p =* 0.0285)
**STRATUS** Dimopolous et al., 2016^14^	III	Pd	682	RRMM	16.8	32.6	4.6	11.9
**OPTIMISMM** Richardson et al., 2019^8^	III	VPd vs. Vd	281 vs. 278	LEN‐pretreated RRMM after 1–3 LOTs	15.9	82.2 vs. 50.0 (*p* < 0.0001)	11.2 vs. 7.10 (*p* < 0.0001)	Not reached
**Alliance A061202** Voorhees et al., 2021^15^	I/II	IPd	29	LEN/PI‐refractory MM	28.4 (I)/26.0 (II)	51.7	4.4	34.3
**NCT01464034** Shah et al., 2015^16^	I	KPd	32	RRMM	26.3	50	7.2	20.6
**NCT01432600** Baz et al., 2016^17^	II	Pd vs. PCd	36 vs. 34	LEN‐refractory RRMM	Not reported	38.9 vs. 64.7 (*p* = 0.035)	4.4 vs. 9.5 (*p* = 0.106)	16.8 vs. not reached
**NCT01754402** Sivaraj et al., 2018^18^	I/II	Benda‐Pd	38	RRMM	17.5	61	9.6	21.3
Dara‐based								
**CASTOR** Sonneveld et al., 2022^19^, ^20^	III	DVd vs. Vd	251 vs. 247	RRMM	72.6			49.6 vs. 38.5 (*p* = 0.0075)
					47	85 vs. 63	16.7 vs. 7.1	
**LEPUS** Fu et al., 2023^21^	III	DVd vs. Vd	141 vs. 70	Chinese patients with RRMM	25.1	84.7 vs. 66.7 (*p* = 0.00314)	14.8 vs. 6.3 (*p* < 0.00001)	Not reported
**POLLUX** Dimopoulos et al., 2023^22^, ^23^	III	DRd vs. Rd	286 vs. 283	RRMM	79.7			67.6 vs. 51.8 (*p* = 0.0044)
					51.3	93 vs. 76	45.8 vs. 17.5 (*p* < 0.0001)	
**PLEIADES** Chari et al., 2020^24^	II	DRd	65	RRMM	14.7	93.8	Not reported	Not reported
**CANDOR** Usmani et al., 2023^25^, ^26^	III	DKd vs. Kd	312 vs. 154	RRMM after 1–3 LOTs	50		28.4 vs. 15.2	50.8 vs. 43.6 (*p* = 0.042)
					17	84 vs. 75 (*p* = 0.008)		
**PLEIDAES** Moreau et al., 2023^27^	II	DKd	66	LEN‐pretreated RRMM after 1 LOT	12.4	84.8	Not reported	Not reported
**EQUULEUS** Moreau et al., 2023^27^	Ib	DKd	85	RRMM after 1–3 LOTs	23.7	81.2	25.7	Not reached
**DARIA** Terpos et al., 2024^28^	II	DId	50	LEN‐pretreated RRMM after 1 LOT	23.4	64	8.1	39.2
**NCT04065308** Byun et al., 2022^29^	II	Dara‐dCEp	32	RRMM with extramedullary disease	11	67.7	5	10
**LYRA** Yimer et al., 2022^30^	II	DVCd	14	RMM	35.3	86	21.7	Not reached
**NCT03314181** Bahlis et al., 2021^31^	I	Ven‐Dd/Ven‐DVd	24/24	t(11;14) RRMM/cytogenetically unselected RRMM	20.9 vs. 20.4	96 vs. 92	Not reached	Not reached
DPd								
**APOLLO** Dimopoulos et al., 2023^11^, ^32^	III	DPd vs. Pd	151 vs. 153	RRMM	39.6			34.4 vs. 23.7 (*p* = 0.2)
					16.9	69 vs. 46 (*p* < 0.0001)	12.4 vs. 6.9 (*p* = 0.0018)	
**MM‐014** Bahlis et al., 2022^33^	II	DPd	112	LEN‐pretreated RRMM after 1–2 LOTs	28.4	77.7	30.8	Not reported
**EQUULEUS** Chari et al., 2017^34^	Ib	DPd	103	RRMM after ≥2 LOTs	13.1	60	8.8	17.5
Sebag et al., 2019^35^	II	DPCd vs. DCd + Pom (only at PD)	120	RRMM	8.2	88.5 vs. 50.8	NR vs. 10.9	Not reached

Abbreviations: Benda, bendamustine; C, cyclophosphamide; D, daratumumab; d, dexamethasone; Dara‐dCEp, daratumumab + cyclophosphamide + cisplatin + etoposide + dexamethasone; E, etoposide; I, ixazomib; K, carlfilzomib; LEN, lenalidomide; P, pomalidomide; R, lenalidomide; V, bortezomib; Ven, venetoclax.

Previous studies revealed encouraging clinical outcomes of Dara‐based regimens (Table 5). Results of the pivotal phase III CASTOR and POLLUX studies reported an ORR of 85% and 93%,^19^, ^22^ respectively, remarkably similar to the observed ORR (84.6%) of the Dara‐based cohort in the present study. However, a relatively inferior median PFS was observed compared to previous trials (10.5 vs. 16.7 months in CASTOR and 45.8 months in POLLUX).^19^, ^22^ This can partly be attributed to biases in population selection between the real‐world context and clinical trial settings. In comparison with our cohort, in which a substantial proportion of patients exhibited treatment refractoriness, patients refractory to PI and lenalidomide were systematically excluded in the CASTOR and POLLUX studies, with the proportions of lenalidomide‐refractory and bortezomib‐refractory patients at merely 24% and 20.6%, respectively.^19^, ^22^ The extended PFS of the non‐lenalidomide‐refractory subgroup of the Dara‐based cohort further validates this explanation (median PFS of 23.9 months). Moreover, the high frequency of dose interruptions (45.6%) in clinical practice potentially attenuated the durability of efficacy. In comparison, a real‐world study from the Mayo Clinic observed worse outcomes (ORR 47%, median PFS of 5.5 months) of Dara combination therapies in a heavily pretreated MM patient population (median LOT of 4).^41^ Nevertheless, our study, despite discrepancies with clinical trial outcomes, indicated that Dara‐based regimens maintained promising efficacy in this Chinese real‐world patient cohort.

Pom‐based and Dara‐based regimens have become common treatment options after the failure of front‐line lenalidomide‐based therapy. However, to the best of our knowledge, there has been a paucity of comparisons made between Pom‐based and Dara‐based regimens for RRMM in either clinical trials or real‐world settings. The present research is the first retrospective cohort study that has compared these two drugs in a real‐world context. On the basis of well‐balanced baseline characteristics, the study showed that Dara‐based regimens yielded a superior overall response (84.6% vs. 57.8%, *p =* 0.002) and ≥VGPR rate (41.5% vs. 15.6%, *p* = 0.004) compared to Pom‐based regimens. Dara‐based regimens were also associated with PFS benefit in the multivariate analysis (HR: 0.46, 95% CI: 0.27–0.79).

However, the results of exploratory subgroup analysis indicated that in patients characterized by poor prognostic features (double‐refractory, multiple relapses [LOT ≥ 2], advanced age, ECOG PS ≥ 2, with extramedullary infiltration), the superiority of Dara‐based therapy was not apparent. In a real‐world study conducted at two centers in the United States that involved 299 patients, a decreasing response rate to Dara with later LOT was observed (1 L 100.0%, 2 L 78.8%, 3 L+ 65.2%).^42^ A retrospective cohort study from the Canadian Myeloma Research Group database (CMRG‐DB) noticed a similar trend of prognosis (median PFS: 2 L 23.5 months, 3 L 12.8 months, 4 L 7.0 months) in 583 MM patients.^43^ Our results, expanding on previous findings, lend support to the hypothesis that administering optimal treatment regimens such as Dara‐based combinations, in the early stage of MM, rather than administering them later, may increase clinical benefit. Indeed, evidence from clinical trials also reflects this notion. The results of CASTOR and POLLUX showed the addition of Dara at first relapse greatly reduced the risk of PD or death by 81% and 58%, respectively.^9^, ^10^ By far, Dara‐based regimens have been recommended as salvage therapy at first relapse by IMWG,^44^ National Comprehensive Cancer Network (NCCN)^45^ and mSMART guidelines.^46^ However, due to the nonrandomized approach, this finding should be interpreted with caution, and head‐to‐head comparisons are still warranted for selecting the optimal treatment sequence.

Moreover, we have also presented the clinical data of 24 patients receiving DPd regimens in our hospital. A median PFS of 6.7 months (95% CI: 4.0–9.3) was reported, which is shorter than the PFS previously reported in clinical trials (12.4 months in APOLLO, 8.8 months in EQUULEUS, and 30.8 months in MM‐014) (Table 5).^11^, ^33^, ^34^ Unlike these clinical trials, our cohort reflects a real‐world unselected patient population, which includes a higher proportion of frail patients (ECOG PS ≥ 2: 37.5% vs. 4% in APOLLO) and patients with refractory status (PI‐refractory: 91.7% vs. 47% in APOLLO).^11^ Despite the failure to observe the response superiority of DPd over Pom‐based therapy (ORR: 75.0% vs. 57.8%, *p =* 0.16; ≥VGPR: 33.3% vs. 15.6%, *p* = 0.09), there was a PFS improvement in the multivariate model (HR: 0.48, 95% CI: 0.24–0.95). This finding suggests the addition of Dara into Pom and dexamethasone regimens may provide prognostic benefit compared to other combination therapies. It is worth noting that this DPd cohort represents a single‐center cohort with a relatively small sample size, which may introduce certain biases in estimating outcomes.

Notably, in the treatment groups containing Dara (Dara‐based and DPd), higher rates of infection were reported. Data from a number of randomized clinical trials also found the addition of Dara to PIs/immunomodulatory drugs increases the risk of infection (upper respiratory tract infection: from 14% to 44% and 23% to 63%),^9^, ^10^, ^11^, ^25^, ^47^ which might be attributable to the immunoparesis induced by the CD38 receptor downregulation and the depletion of CD38‐expressing immune cells.^48^ Our results highlight the need to optimize monitoring, prophylaxis and supportive care in clinical practice for Dara‐related infections.

This research is limited by its single‐center retrospective design. However, the baseline characteristics were generally balanced between the patient groupings, and multivariate analyses and subgroup analyses were undertaken to mitigate potential bias. Due to the recent approval of Pom and Dara in China, the sample size was relatively small. The relatively small sample size, particularly in certain subgroups such as LEN‐non‐refractory and DPd groups, may have limited the statistical power to detect subtle differences or associations. The dosage and drugs used were not uniform, which might have impacted the evaluation of clinical outcomes. Some patients were followed‐up in the outpatient setting, which may have resulted in loss of information. In addition, we failed to make a robust assessment of OS because of the short follow‐up time.

Nevertheless, this study provides valuable real‐world comparative information between Pom‐based, Dara‐based, and DPd regimens for the treatment of RRMM populations. Considering the limitations of retrospective studies, more well‐designed multicenter randomized clinical trials with larger sample sizes are highly anticipated to guide clinical decision‐making.

## 5 CONCLUSIONS

Pom‐based, Dara‐based, and DPd therapies had favorable clinical efficacies in patients with RRMM. Dara‐based regimens were associated with improved outcomes compared to Pom‐based therapies, albeit the superiority was not evident among patients with poor prognostic features. The DPd regimen also led to prolonged PFS compared to Pom‐based therapies. Concerning safety, Dara‐related infections warrant greater emphasis in clinical practice.

## AUTHOR CONTRIBUTIONS

**Xiaoyan Han:** Conceptualization (equal); funding acquisition (lead); project administration (equal); resources (equal); supervision (equal); writing – original draft (equal); writing – review and editing (lead). **Xincheng Jiang:** Conceptualization (equal); data curation (equal); investigation (equal); methodology (equal); software (equal); validation (equal); visualization (equal); writing – original draft (lead); writing – review and editing (equal). **Jingsong He:** Conceptualization (equal); resources (equal); writing – review and editing (equal). **Gaofeng Zheng:** Conceptualization (supporting); resources (equal); writing – review and editing (equal). **Yaqin Xiong:** Conceptualization (supporting); data curation (equal); writing – review and editing (supporting). **Yanling Wen:** Project administration (supporting); resources (equal); writing – review and editing (supporting). **Yang Yang:** Resources (equal); writing – review and editing (supporting). **Donghua He:** Resources (equal); writing – review and editing (supporting). **Qingxiao Chen:** Resources (equal); writing – review and editing (supporting). **Yi Zhao:** Resources (equal); writing – review and editing (supporting). **Yi Li:** Resources (equal); writing – review and editing (supporting). **Wenjun Wu:** Resources (equal); writing – review and editing (supporting). **Zhen Cai:** Conceptualization (equal); funding acquisition (lead); project administration (lead); resources (lead); supervision (equal); writing – review and editing (equal).

## FUNDING INFORMATION

This work was funded by the National Natural Science Foundation of China under Grant number 82270205.

## CONFLICT OF INTEREST STATEMENT

The authors have no relevant financial or non‐financial interests to disclose.

## ETHICS APPROVAL

The study was conducted in accordance with the Declaration of Helsinki and was approved by the Ethics Committee of the First Affiliated Hospital, Zhejiang University School of Medicine, Hangzhou, Zhejiang, China, with an exemption from informed consent (Reference Number: 20230318). No specific consent is needed for statistical analyses of aggregated de‐identified data. For this study, the raw data were first extracted from HIS, and patients' identities, including names, screening IDs, patient IDs, and mobile phone numbers, were de‐identified.

## CONSENT FOR PUBLICATION

Not applicable.

## Supporting information


Tables S1‐S3.

## Data Availability

The datasets used and/or analyzed during the current study are available from the corresponding author on reasonable request.
