# Supplementary material for: Clinical outcomes of pomalidomide‐based and daratumumab‐based therapies in patients with relapsed/refractory multiple myeloma: A real‐world cohort study in China
Source: Cancer Med. 2024 May 2;13(9):e7232. doi: 10.1002/cam4.7232 (PMC11066492; doi:10.1002/cam4.7232)
Supplement: Supplementary file 1 — Tables S1‐S3. [file CAM4-13-e7232-s001.docx]

Table S1. Treatment protocols

| **Protocol** | **Number (%)** |
| --- | --- |
| Pom-based regimens | 48 |
| VPd | 24 (50.0) |
| IPd | 12 (25.0) |
| PCd | 6 (12.5) |
| Pd | 6 (12.5) |
| Dara-based regimens | 68 |
| DVd | 27 (39.7) |
| DRd | 23 (33.8) |
| D-selinexor-d | 17 (25.0) |
| D-chemotherapy* | 1 (1.5) |
| DPd regimens | 24 |
| DPd | 22 (91.7) |
| DVPd | 2 (8.3) |

Data are shown as n (%). *D-chemotherapy refers to daratumumab+ cisplatin+ etoposide+ ifosfamide+ dexamethasone. V, bortezomib. D, daratumumab. P, pomalidomide. d, dexamethasone. I, ixazomib. C, cyclophosphamide. R, lenalidomide.

Table S2. Detailed response of patients treated with Pom-based, Dara-based, and DPd regimens

| Responses | Pom-based  (n = 48) | Dara-based  (n = 68) | DPd  (n = 24) | *P*  value | *P_1_^*^* value | *P_2_*^†^ value |
| --- | --- | --- | --- | --- | --- | --- |
| Evaluable for response, n | 45 | 65 | 24 |  |  |  |
| ORR | 26 (57.8) | 55 (84.6) | 18 (75.0) | **0.007** | **0.002** | 0.16 |
| ≥ VGPR | 7 (15.6) | 27 (41.5) | 8 (33.3) | **0.02** | **0.004** | 0.09 |
| ≥ CR | 2 (4.4) | 11 (16.9) | 5 (20.8) | 0.09 |  |  |
| VGPR | 5 (11.1) | 16 (24.6) | 3 (12.5) |  |  |  |
| PR | 19 (42.2) | 28 (43.1) | 10 (41.7) |  |  |  |
| MR | 6 (13.3) | 3 (4.6) | 3 (12.5) |  |  |  |
| SD | 11 (25.0) | 6 (9.2) | 3 (12.5) |  |  |  |
| PD | 2 (4.4) | 1 (1.5) | 0 (0.0) |  |  |  |

Data are shown as n (%) unless otherwise stated. **P_1_* value: comparison between Pom-based and Dara-based group. ^†^*P_2_* value: comparison between Pom-based and DPd group. ORR, overall response rate. CR, complete response. VGPR, very good partial response. PR, partial response. MR, minimal response. SD, stable disease. PD, progression disease. Pom-based, pomalidomide-based. Dara-based, daratumumab-based. DPd, daratumumab plus pomalidomide and dexamethasone.

| **Parameter** | **Univariate OR for ORR (95% CI)** | ***P* value** | **Univariate HR for**  **PFS (95% CI)** | ***P* value** |
| --- | --- | --- | --- | --- |
| Treatment (Pom-based reference) |  |  |  |  |
| Dara-based | 4.02 (1.64-9.85) | **0.002** | 0.56 (0.34-0.93) | **0.02** |
| DPd | 2.19 (0.73-6.57) | 0.16 | 0.61 (0.31-1.18) | 0.14 |
| Age (≤ 65 vs > 65) | 1.30 (0.60-2.85) | 0.51 | 1.18 (0.75-1.87) | 0.47 |
| Extramedullary disease (no vs yes) | 1.20 (0.49-2.98) | 0.69 | 1.28 (0.78-2.10) | 0.33 |
| DS (I+II vs III) | 0.40 (0.13-1.26) | 0.12 | 1.43 (0.79-2.61) | 0.24 |
| ISS (I+II vs III) | 0.90 (0.41-1.97) | 0.80 | 1.06 (0.67-1.67) | 0.82 |
| Cytogenetics (no high risk or NA vs high risk) | 0.63 (0.29-1.36) | 0.24 | 1.35 (0.86-2.13) | 0.20 |
| eGFR  (< 60 vs $\geq$60 ml/min) | 1.47 (0.64-3.36) | 0.37 | 0.81 (0.50-1.31) | 0.38 |
| ECOG PS (0-1 vs $\geq$2) | 0.66 (0.28-1.55) | 0.34 | 1.96 (1.17-3.30) | **0.01** |
| Prior antimyeloma  lines of therapy | 1.04 (0.71-1.50) | 0.86 | 0.87 (0.69-1.10) | 0.25 |
| Lenalidomide refractory  (no vs yes) | 0.55 (0.22-1.39) | 0.21 | 1.71 (0.97-2.99) | **0.06** |
| PI refractory (no vs yes) | 0.92 (0.39-2.15) | 0.85 | 1.09 (0.66-1.78) | 0.74 |
| Double refractory  (no vs yes) | 0.74 (0.34-1.60) | 0.44 | 1.38 (0.87-2.19) | 0.18 |

Table S3. Univariate analysis OR for ORR and HR for PFS

HR (95% CI) and OR (95% CI) have been rounded to the nearest hundredth. HR, hazard ratio. OR, odds ratio. CI, confidence interval. Dara-based, daratumumab-based. DPd, daratumumab plus pomalidomide and dexamethasone. DS, Durie-Salmon Staging System. ISS, International Staging System. NA, not available. eGFR, estimated glomerular filtration rate by Cockcroft-Gault equation. ECOG PS, Eastern Cooperative Oncology Group performance status. PI, proteasome inhibitor.
